# Not all steps are equal: independent prospective associations of stepping volume and patterns with incident type 2 diabetes mellitus in the Maastricht study

**DOI:** 10.1186/s12966-025-01839-z

**Published:** 2025-11-19

**Authors:** Richard M. Pulsford, Esmée A. Bakker, Matthew Ahmadi, Joanna M. Blodgett, Hans Bosma, Laura Brocklebank, Simone JPM Eussen, Mark Hamer, Rebecca Lear, Brad Metcalf, Hans Savelberg, Emmanuel Stamatakis, Annemarie Koster

**Affiliations:** 1 Faculty of Health and Life Sciences, University of Exeter, Exeter, UK; 2 Department of Medical Biosciences, Radboud university medical center, Nijmegen, Netherlands; 3 Department of Physical Education and Sports, Sport and Health University Research Institute (iMUDS), University of Granada, Granada, Spain; 4 Department of Primary and Community Care, Radboud university medical center, Nijmegen, Netherlands; 5 Mackenzie Wearables Research Hub, Charles Perkins Centre, The University of Sydney, Sydney, NSW Australia; 6 School of Health Sciences, Faculty of Medicine and Health, The University of Sydney, Sydney, NSW Australia; 7 Institute of Sport, Exercise and Health, Division of Surgery and Interventional Sciences, London, UCL UK; 8 University College London Hospitals NIHR Biomedical Research Centre, London, UK; 9 Department of Social Medicine, CAPHRI Care and Public Health Research Institute, Maastricht University, Maastricht, Netherlands; 10 Nuffield Department of Population Health, Big Data Institute, University of Oxford, Old Road Campus, OX3 7LF, Oxford, UK; 11 Department of Epidemiology, CAPHRI Care and Public Health Research Institute and CARIM Cardiovascular Research Institute Maastricht, Maastricht University, Maastricht, Netherlands; 12 Department of Nutrition and Movement Science Maastricht University, Maastricht, Netherlands

**Keywords:** Accelerometers, ActivPal, Daily steps, Physical activity, Activity patterns, Type 2 diabetes, Prospective study

## Abstract

**Background:**

Stepping has been associated with reduced risk for type 2 diabetes (T2D), but existing prospective studies focus largely on average stepping volume (steps per day or week) and ignore important differences in how stepping is accumulated. Here, we examined independent associations of stepping volume and within and between day variability, with incident T2D.

**Methods:**

Participants (*n* = 4594, 40-75y) without preexisting T2D from The Maastricht Study wore an activPAL3 accelerometer (6–7 days). Prospective associations of stepping volume (steps/day) with incident T2D were assessed using Cox proportional hazards models with restricted cubic splines, adjusted for age, sex, BMI, education, smoking, CVD, sedentary time and diet. Four indices of between-day (i-iii below) and within-day (iv below) stepping pattern were modelled alongside total steps/day. These were: (i) proportion of steps accumulated on the 2 most active days (%Active-2days), (ii) between-day step count variability (BDV) and (iii) inter-daily step count stability (IS), (iv) within-day variability in stepping (WDV) (variability in steps/hour). Higher values in %Active-2days, BDV and WDV indicate greater variation in stepping between or within days. Higher IS values indicate greater uniformity in hourly stepping pattern between days.

**Results:**

Over 30,336 person-years of follow-up (mean 6.6y), 178 incident cases of T2D were recorded. A non-linear (*p* = 0.04) ‘L-shaped’ association was observed between stepping volume and T2D risk, with steeper risk reduction earlier in the steps/day distribution. Relative to accumulating ≤ 5000 steps/day, adjusted hazard ratios (95% CI) were 0.57 (0.34, 0.96) for 5000–7500 steps/day, 0.60 (0.65–0.94) for 7501–10,000 steps/day, 0.48 (0.25, 0.89) for 10,001–12,500 steps/day and 0.68 (0.37, 1.24) for > 12,501 steps/day. Higher %Active-2days, BDV, and lower IS, (cumulatively describing a stepping pattern which is variable between days and within days), were linearly associated with T2D risk independent of stepping volume. HRs per SD increase were: %Active-2days 0.70 (0.65, 0.97), BDV 0.69 (0.54, 0.89) and IS 1.32 (1.08, 1.63).

**Conclusions:**

Substantial reductions in T2D risk can be achieved by accumulating more steps during the day. Further, accumulating steps in a pattern possibly reflecting periodic larger doses of stepping may provide additional reductions in T2D risk. Future research regarding volume and optimum patterns of stepping could form the basis of the next generation of public health guidance and interventions to improve health through movement.

**Supplementary Information:**

The online version contains supplementary material available at 10.1186/s12966-025-01839-z.

## Background

Prevalence of type 2 diabetes (T2D) is growing, affecting over 96% of the estimated 529 million people living with diabetes worldwide [1]. The 2016 Non-Communicable Disease Risk Factor Collaboration (NCD-RisC) Study projected the probability of meeting global targets to halt the rise in diabetes prevalence to be < 1% [2]. Understanding modifiable risk factors and effective strategies to reduce T2D remains a global public health priority.

Physical activity (PA) improves glucose homeostasis acutely via insulin-independent clearance of circulating glucose, and chronically via improvements in insulin sensitivity and body composition [3, 4]. Prospective studies suggest that higher PA levels can reduce risk of incident T2D [5–7]. Public health guidelines recommend that adults achieve a minimum of 150 min per week of moderate-vigorous PA (MVPA) to reduce the risk of chronic non-communicable conditions such as diabetes [8, 9]; however global prevalence of PA at this recommended level remains low [10].

Stepping, encompassing walking, running and stair climbing, is a fundamental PA behaviour that is associated with favourable health outcomes [11–13]. The proliferation of wearable activity trackers that can measure free-living stepping across multiple days presents new opportunities for research [14]. In addition, the ubiquitous capture of stepping by smart watches and smart-phone applications offers significant potential for population level insights into day-to-day stepping patterns and as a platform for interventions [15]. At present no national or international physical activity guidelines include recommendations for stepping. While different devices used in different ways may capture stepping slightly differently (due to differences in hardwear, on-board processing and wear location amongst other factors), stepping-based PA metrics have promise as the basis of future public health guidelines due to their simplicity, interpretability, and the accessibility of self-monitoring [16]. 

To date, a small number of existing prospective studies have examined associations between stepping and incident T2D [17–20]. While these studies provide insight into associations of average daily stepping volume (steps/day) with incident T2D risk, they largely do not consider potentially important differences in the daily and weekly patterns in which stepping is accumulated. The importance of differences in stepping pattern for T2D risk is intuitive. A high weekly volume of steps may be accumulated rapidly on a few days via relatively short periods of exercise; which is known to reduce diabetes risk via improvements in insulin sensitivity and beneficial changes in body composition [3]. Conversely, a similar high volume of steps may be accumulated across much longer periods as part of daily occupational activity, but this may not confer the same health benefits [21, 22]. 

There is evidence that differences in between-day and within-day patterns of PA accumulation can influence health over and above total PA volume [23, 24]. Two studies have reported that neither the variability nor stability of accelerometer-based estimates of daily energy expenditure were associated with T2D risk [25, 26]. However, there is growing evidence for associations between volume-independent differences in daily stepping patterns and diabetes risk factors, including age, BMI and vascular function [23, 27, 28]. 

The importance of different between- and within-day patterns of step accumulation for T2D risk remains poorly understood but may provide opportunities for more detailed public health guidance, or interventions which could focus on optimising how a given stepping volume is achieved rather than focussing only on stepping more. The aim of this study is to examine how stepping volume and variations in stepping volume between- and within-days are associated with risk of T2D.

## Methods

### Study design and participants

The Maastricht study is a prospective population-based cohort study of adults living in the southern Netherlands. The rationale and methodology of The Maastricht study have been described previously [29]. Briefly, the Maastricht study focuses on the etiology, pathophysiology, complications, and comorbidities of T2D and is characterized by an extensive phenotyping approach. Eligible participants were between 40 and 75 years of age and were recruited through mass media campaigns, from the municipal registries and the regional Diabetes Patient Registry via mailings. Recruitment was stratified according to known diabetes status, with an oversampling of individuals with T2D. The present analysis includes data on participants without T2D, drawn from the full Maastricht cohort of 9187 participants, who completed the baseline survey between November 2010 and October 2020. The examinations of each participant were performed within a time window of three months. The study was approved by the institutional ethical committee (NL31329.068.10) and the Minister of Health, Welfare, and Sports of the Netherlands (permit no. 131088-105234-PG). All participants gave written informed consent.

### Ascertainment of incident T2D

Diabetes status at baseline was assessed and classified based on a standardised oral glucose tolerance test following an overnight fast, and medication use according to the World Health Organisation 2006 criteria [29]. Participants with T2D at baseline were excluded. Participants on diabetes medication and without type 1 diabetes were also considered to have T2D and also excluded.

Diabetes Survival Time after baseline was assessed by annual self-report. Participants were asked “Has a doctor told you that you have diabetes in the last 12 months with possible responses “yes”, ”no” or ”I don’t know” ("I don’t know" was coded as a missing value). This measure has been validated against reference diabetes indices, including fasting glucose and medication use [30]. Survival time (years) for diabetes cases was computed as the midpoint between the follow-up date at which the event was reported and the previous follow-up date. Survival time (years) for censored cases was computed as the time of the last available follow-up date.

### Assessment of daily stepping volume and pattern

Stepping was assessed using the activPAL3™ PA monitor (PAL Technologies, Glasgow, UK). The activPAL3 is a small (53 × 35 × 7 mm), lightweight (15 g) triaxial accelerometer that records movement in vertical, anteroposterior and mediolateral axes, and determines posture (sitting or lying, standing and stepping) based on movement acceleration and inclinometer orientation. The device was placed in a waterproof nitrile sleeve and then attached directly to the skin on the anterior aspect of the right thigh with a waterproof adhesive dressing (3 M Tegaderm™). Participants were asked to wear the accelerometer continuously for eight complete (24 h) consecutive days. Participants were asked not to replace the device once removed. Data were uploaded using manufacturers software and estimates of accelerometer wear and stepping computed using customised software written in MATLAB R2018b (MathWorks, Natick, MA, USA) https://www.demaastrichtstudie.nl/research/accelerometry. Data from the first day were excluded from the analysis of PA to exclude data recorded during laboratory testing procedures undertaken after attachment of the device. Participants were included in the present analysis if they provided at least 6 complete days (a complete day being 24 h or accelerometer wear starting at midnight).

Stepping volume was quantified as the average daily step count recorded over the wear period. Between and within day variability in stepping accumulation were captured as described below. Examples of how a given volume of stepping can be accumulated in different between and within day patterns are described in supplementary figures S1 a and b [23].

### Variation in step count between and within days

- The proportion of step count accumulated on the two most active days of the week (%Active-2days): Daily step count was expressed as a percentage of weekly step count and the two most active days summed to calculate %Active-2days. This is similar to the ‘weekend warrior’ profile described elsewhere [31], although here retained as a continuous exposure measure rather than dichotomising participants into those who achieved above or below 50% of activity in the most active 2 days. For participants in the analytical sample with 6 complete days of accelerometer wear the average daily step count was multiplied by 7 to allow the 2 most active 7 days for all participants to be represented as a proportion of 7 days of stepping.
- Between-day step count variability (in steps, BDV): Calculated as the standard deviation (SD) of the differences in total step count between consecutive measured days.
- Inter-daily stability in step count (IS): The extent to which the pattern of hourly step counts is consistent across days, represented by the ratio (0–1) of the difference between each hourly step count and the sample mean step count for that hour. 1 indicates perfect stability (consistent hour-by-hour stepping pattern across days), while zero indicates a pattern that is inconsistent between days.
- Within-day step count variability (WDV): Calculated as the SD of differences between step count for consecutive hours of each consecutive day.

These metrics capture simple variability of stepping between and within days and have been used previously to examine associations with diabetes risk and other health outcomes [23, 26, 32]. Mathematical formulae for these metrics of activity variability have been detailed previously [33], and their application to stepping data from The Maastricht Study is described by Lear et al. [23]

### Covariates

Covariates included age (years), sex, educational level (low, medium and high), BMI (kg/m^2^), smoking status (never, former smoker, current smoker) and sedentary time. Habitual dietary quality was calculated using a validated food frequency questionnaire [34] and quantified using the Dutch Healthy Diet Index [35]. Prevalent cardiovascular disease was defined as a self-reported history of myocardial or cerebrovascular infarction or percutaneous artery angioplasty of, or vascular surgery on, the coronary, abdominal, peripheral, or carotid arteries [36]. 

### Statistical analyses

Analyses were conducted in Stata (V17. StataCorp, College Station Texas, US) and R (V4.2.3. R Core Team, Vienna, Austria). Prospective associations of stepping with incident T2D were assessed using Cox Proportional Hazards models with restricted cubic splines. We tested restricted cubic splines with 3 knots (located at 10th, 50th, and 90th centiles), 4 knots (at 5th, 35th, 65th, and 95th centiles), and 5 knots (at 5th, 27.5th, 50th, 72.5th, and 95th centiles) and calculated the Akaike information criterion to identify the best fit model [37]. In case of a non-linear association, a likelihood ratio test was used to test whether additional knots significantly improved the model. When the model improvement was not significant the model with a lower number of knots was used for the main analyses. Analytical models were adjusted for age, sex, BMI, education and accelerometer wear time (model 1), and subsequently for smoking status, Dutch Healthy Diet index, CVD and sedentary time (model 2). To examine the volume-independent associations of the 4 stepping pattern metrics, these were added individually in models 3a-d. Pearson coefficients examined the correlations between stepping volume and pattern metrics. To examine whether associations between average daily step count and incident T2D differed between a priori defined subgroups, interaction terms were fitted for step count with sex and age. To examine whether the association between PA pattern metrics and incident T2D differed according to stepping volume, an interaction term was fitted for average daily step count. Proportional Hazards assumptions were assessed using a test of Schoenfeld residuals and Nelson-Aelen plots.

### Sensitivity analyses

To explore the possible impact of the number of knots on dose response associations between stepping volume and diabetes we performed sensitivity analyses using 4 and 5 knots. Analyses were repeated with additional adjustment for within-day step count variability for examination of between-day step count variability, and vice versa. Analyses were also repeated with; (a) household-adjusted income and (b) occupational status (based on ISEI-08 classification) as indicators of socioeconomic position instead of education level. Analyses were repeated with further adjustment for family history of T2D, and for mobility limitations determined using the 36-item Short-Form Health Survey (SF-36) [38] and defined as having difficulty walking 500 m and/or climbing up a flight of stairs, to explore its role as a confounder, or on the causal pathway.

To explore the potential confounding effects of occult diabetes at baseline, analyses were repeated following exclusion of incident T2D cases within the first 12 months of follow-up. Analyses were also repeated with survival time calculated as time from baseline assessment to the midpoint between reporting of diabetes and the event of the preceding follow-up questionnaire (conservative method).

## Results

### Participants

The final analytical sample consisted of 4594 participants without T2D at baseline who wore an accelerometer for ≥ 6 days, and had complete data for stepping and covariates (see Supplementary Figure S2). Sample characteristics are described in Table 1. Those in the sample were slightly older on average and had a slightly better Dutch Healthy Diet Index score than those who were excluded. There were no differences by sex, educational level, smoking status, BMI, waist circumference or HbA1C. A total of 178 incident T2D cases were recorded over 30,336 person-years of follow-up (mean follow-up time 6.6 ± 2.6 years).

**Table 1:** Participant characteristics

		Total sample		Males		Females	
N (%)		4594		2014	(44%)	2580	(56%)
Age (years)		59.50	(8.5)	60.60	(8.41)	58.65	(8.55)
Education level (%)	Low	30.13		25.18		33.95	
	Intermediate	27.40		25.64		28.76	
	High	42.47		49.18		37.28	
BMI (kg/m 2)		25.89	(3.82)	26.37	(3.32)	25.52	(4.13)
Waist Circ. (cm)		91.48	(11.81)	97.49	(10.04)	86.86	(10.97)
HbA1C (mmol/mol)		35.72	(4.79)	35.79	(4.90)	35.65	(4.69)
Smoking Status (%)	Never	41.20		38.79		43.07	
	Former	47.83		49.54		46.51	
	Current	11.67		11.67		10.42	
Dutch Healthy Diet Score		85.61	(15.02)	80.78	(15.26)	89.36	(13.71)
Steps/day		10,028	(3504)	10,063	(3695)	9987	(3350)
Sedentary time	hrs/day	9.13	(1.56)	9.63	(1.52)	8.74	(1.49)

Table 1. Participant characteristics. Data are mean ± SD unless otherwise stated. Education was reported in the Maastricht study in the following categories: 1 = None, 2 = Primary educational level, 3 = Lower vocational education, 4 = Intermediate general secondary education, 5 = Intermediate vocational education, 6 = Higher general secondary education, 7 = Higher vocational education, 8 = University education,. For categorical analyses these groups were collapsed into Low (groups 1–4– above) Intermediate (5 & 6) and High (7 & 8). The analytical sample described here were slightly older, had a better Dutch Healthy Diet Score than the wider population of DMS. There were no other observable differences between the analytical sample and the wider Maastricht study population.

### Dose-response association between stepping volume and incident T2D

**Fig. 1:**
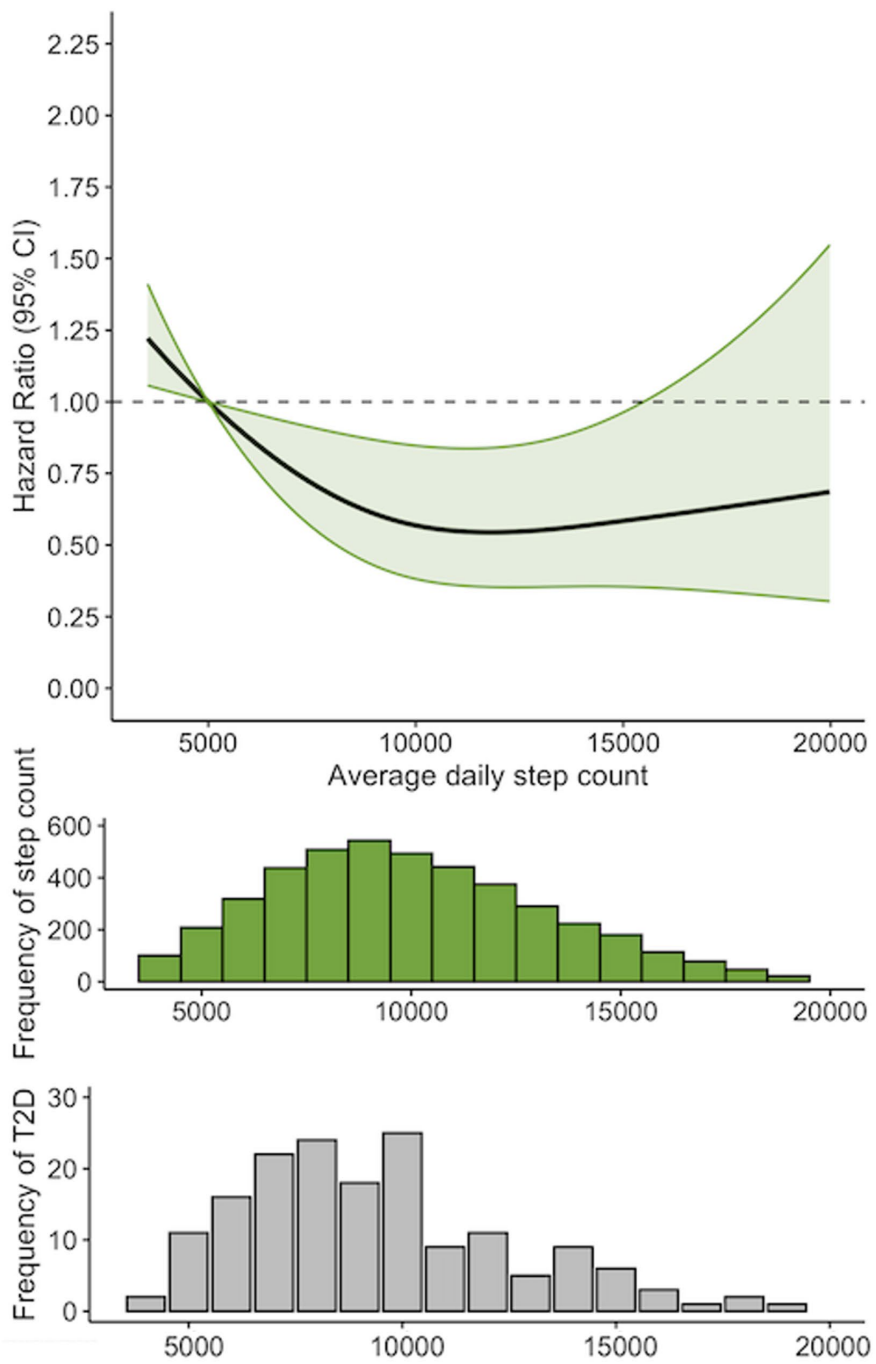
Figure 1 edited for proofs

**Fig. 2:**
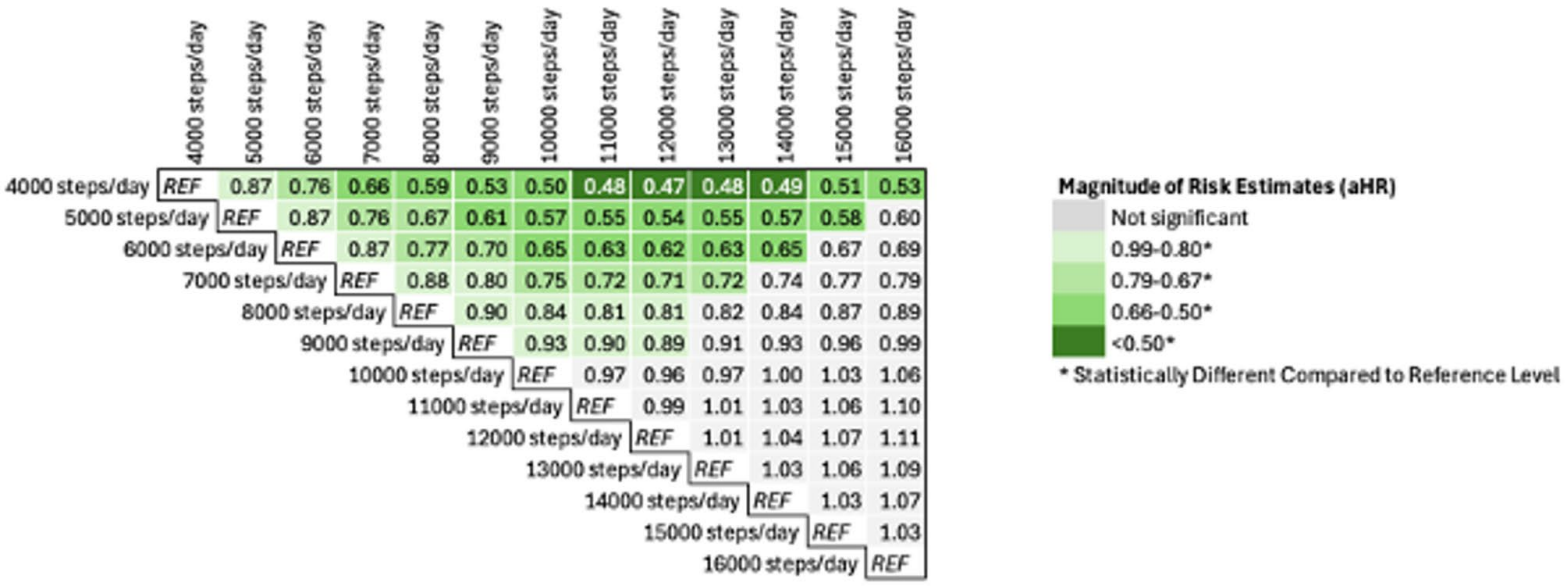
Each row provides estimated Hazard Ratios for 1000 steps/day increments relative to different reference stepping levels (*REF*). Analyses are based on restricted cubic spline models adjusted for age, sex, educational level, smoking status, diet, body mass index (BMI) CVD and daily sedentary time

Schoenfeld residuals and Nelson-Aelen plots did not suggest evidence for any deviations from proportionality in any of the Cox models. We observed a non-linear (*p* = 0.04) association between average steps/day and risk for incident T2D in both minimally adjusted and fully adjusted models. Figure 1 describes the trajectory of diabetes risk according to stepping volume following adjustment for confounders (model 2). Restricted spline models with 3 knots were used as additional knots did not significantly improve the model fit (difference in AICs was nonsignificant with *p* > 0.5). Relative to accumulating 5000 steps/day, hazard ratios for T2D were progressively lower with increasing daily steps before the curve shallowed at approximately 9000 steps/day. Nadir (i.e. lowest point on the risk curve) was at ~ 12,000 steps/day where risk for incident T2D was ~ 46% lower than at 5000 steps/day. Relative to accumulating ≤ 5000 steps/day, adjusted hazard ratios and 95% confidence intervals (95%CI) were 0.57 (0.34, 0.96) for 5000–7500 steps/day, 0.60 (0.65–0.94) for 7501–10,000 steps/day, 0.48 (0.25, 0.89) for 10,001–12,500 steps/day and 0.68 (0.37, 1.24) for > 12,501 steps/day (Table 2). Adjusted hazard ratios for 1000-step increments relative to different reference stepping levels are described in Fig. 2. There were no significant interactions of stepping volume with age or sex (*p* > 0.05). Observed dose-response associations did not differ using restricted cubic spline models with 4 and 5 knots.

**Table 2:** Hazard ratios for incident diabetes according to daily stepping

	n/cases	Person-years	Rate	Hazard Ratios^b^	95% CI
		(x1000)	(/1000 Person Year)		
Average daily steps^a^					
≤ 5000	297/21	1.26	16.55	1	1
5001 to 7500	900/50	5.79	8.64	0.57	0.34, 0.96
7501 to 10,000	1326/57	8.76	6.50	0.60	0.35, 0.94
10,001 to 12,500	1052/22	7.22	3.04	0.48	0.25, 0.89
> 12,501	1019/28	6.99	4.00	0.68	0.37, 1.24
*P* *trend*				*P* < 0.001	

Hazard Ratios and 95% confidence intervals (95% CI) for associations of stepping volume with incident T2D. ^a^ determined by activPal accelerometer worn on the anterior aspect of the mid-thigh for ≥ 6 days; ^b^ adjusted for age, sex, education level, and accelerometer wear time, smoking status, healthy diet score, body mass index (BMI) CVD and daily sedentary time

### Volume-independent associations of between- and within-day stepping pattern with incident T2D

**Fig. 3:**
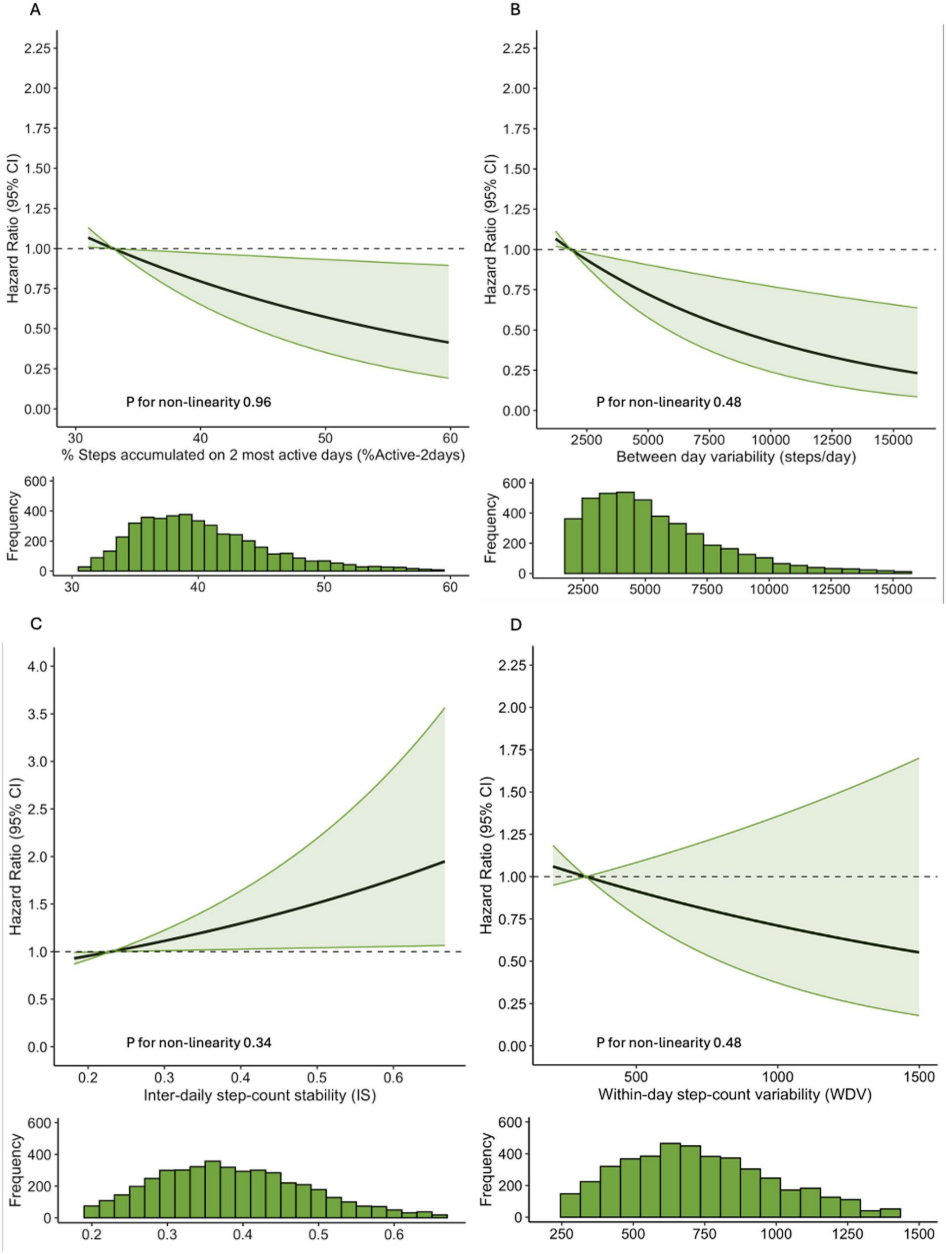
Panels **A**, **B**, **C** and **D**. Volume independent associations of between and within day stepping pattern metrics with incident T2D. The solid line and shaded area represents estimated Hazard ratios and associated 95% confidence intervals (95% CI) for incident T2D according to measures of between day variability in step count following adjustment for covariates (age, sex, educational attainment, smoking status, diet, body mass index (BMI), CVD and daily sedentary time) and also including total stepping volume. (**A**) Proportion of step count accumulated on two most active days, (**B**) Between day variability in step count (see notes above), (**C**) Within-day variability in step count and (**D**) Between day stability in step count

Correlations between stepping volume and pattern metrics are described in supplementary table S1. Only within-day stepping variability was strongly correlated with stepping volume (*r* = 0.7). After adjustment for stepping volume, we observed linear associations between the other 3 of the 4 stepping variability metrics with T2D risk (p values for non-linearity 0.34–0.96). Stepping volume-independent associations between stepping pattern metrics and diabetes risk are described in Fig. 3a and d. Both %Active-2days and BDV were inversely associated with T2D risk. The hazard ratio for an increase of 1 standard deviation in %Active-2days of (SD equal to a 5.7% increase in %Active-2days) was 0.79 (0.65, 0.97) and for BDV (SD equal to BDV of 3106 steps) was 0.69 (0.54, 0.89). Conversely IS was positively associated with T2D risk: hazard ratio per standard deviation increase (SD equal to 0.1) was 1.32 (1.08, 1.63). Collectively a higher %Active-2days and BDV and a lower IS describing a more variable activity pattern. WDV was not associated with T2D risk.

Mutual adjustment for between- and within-day stepping variability, additional adjustment for mobility limitations, adjustment for SEP using alternative indices, family history of diabetes, and survival time using the ‘conservative method’ did not materially impact the findings described. Analyses repeated following exclusion of cases identified within the first 12 months of follow-up are described in Supplementary Fig. 3 (68 cases excluded). Associations were attenuated (slightly smaller hazard ratios and wider confidence intervals) but trends remained comparable. Associations with WDV remained null.

## Discussion

In these prospective analyses of over 4500 people, we examined the independent associations of both device-measured stepping volume and indices describing daily and weekly stepping patterns with incident T2D. After adjustment for important confounding factors and up to 10 years of follow-up (33,600 person-years of follow-up), we observed a strong non-linear inverse association between average daily step count and risk for T2D. Accumulating 12,000 steps/day was associated with 46% lower risk of T2D risk compared to 5000 steps/day. Moreover, an important and novel finding of this work is that the higher proportion of steps accumulated during the two most active days and higher variability of stepping volume between days were associated with lower incident T2D, independent of total stepping volume. Greater stability of stepping patterns between days was associated with higher diabetes risk. These findings suggests that small increases in daily steps have the potential to improve health, but importantly, not all steps are equal: the way in which steps are accumulated may influence the impact on diabetes risk.

The observed inverse association between stepping volume and incident T2D is consistent with previous prospective studies [17–19]. The underpinning physiological mechanisms are intuitive, given that higher PA levels promote better glucose homeostasis [39]. Muscular contraction during movement facilitates insulin-independent glucose transport from the circulation to skeletal muscle via translocation of glucose transporters (e.g. glucose transporter protein type 4: GLUT4) to the cell membrane [3, 40]. More chronic adaptations to PA include changes in muscle fibre type, mitochondrial enzyme content, and an increase in GLUT4, all of which support better glucose transport, and reduced insulin demand [41]. In addition, higher daily stepping may reduce adiposity and inflammatory markers, which are risk factors for T2D [39]. 

Previous studies report inverse linear associations between stepping volume and diabetes risk [17–19]. Here we observed a steep decline in diabetes risk with higher daily stepping volume until 9000 steps/day where the rate of decrease in risk began to slow, and nadir at ~ 12,000 steps/day. Ballin and co-workers [20] observed a similar non-linear inverse relationship between stepping and diabetes risk in a prospective study of community-dwelling older adults in the Swedish Healthy Aging Initiative, where initial steep reduction in risk shallowed at around 6000 steps/day and plateaued at ~ 8000 steps/day. Further, the magnitude of risk reduction associated with increased stepping in the current study (~ 45% for 12000 steps/day compared to 5000 steps/day) is greater than has been reported previously [17–20], albeit comparisons between studies are complicated by differences in study populations (younger and healthier in the present analyses) and the use of different reference levels and devices used to capture stepping.

The observation that the proportion of steps accumulated on the two most active days, higher between-day variability and lower inter-daily stability were all associated with lower diabetes risk after adjusting for stepping volume is an important and novel finding of this work. Collectively these characteristics describe an activity profile in which daily organisation and ‘doses’ of PA vary across days of a week. To date, no existing studies have examined the volume-independent effects of differences in between- and within-day stepping patterns on diabetes risk. Findings from the limited research examining the independent effects on diabetes risk of differences in PA patterns based on other indices of PA are inconsistent. Both Tian et al. [25] and Wu et al. [26] observed that in separate studies of participants from UK Biobank, neither consistency of PA (SD of daily PA energy expenditure in MET-hrs per week) [25] nor IS (based on PA time in minutes) [26] were associated with incident T2D risk. These contrasting findings may reflect differences in the respective study’s population, (the present sample were slightly younger) although important differences in the movement parameters studied make direct comparison problematic. Stepping is a behavioural volume metric and not directly comparable with PA time (in Wu et al. [26], which is finite and does not reflect volume), or estimates of energy expenditure based on movement (in Tian et al. [25], which may reflect variations in movement intensity not captured here).

More broadly, evidence for associations between the variability and stability of PA with health outcomes is inconsistent. In contrast to the present study, more regular and stable activity patterns have previously been associated with lower mortality and CVD risk, hypertension and obesity [42], although, associations were not adjusted for PA volume preventing insight into the independent importance of PA patterns. Studies of older populations in particular have observed health benefits associated with more consistent and stable activity patterns, however it is possible that the regularity of behavioural patterns may be sensitive to different aspects of lifestyle and behaviour at different points on the life-course [24]. Previous studies in similar aged populations have observed similar, more-variable and less-stable PA patterns are associated with better health [23, 24]. 

A possible explanation for more variable stepping patterns being associated with reduced T2D risk may be the contribution of exercise to both variability in daily stepping and in reducing T2D risk. Exercise is likely a key driver of daily and weekly PA patterns [23, 32]. During exercise, a large volume of steps may be accumulated in bouts of a longer duration or at a higher rate. Unless very similar exercise is undertaken at the same time on each day (uncommon at a population level), exercise will result in a stepping profile that varies between and within days, characterised by a high IV, lower IS, and a greater proportion of activity on some days than others. These irregular larger ‘doses’ of activity are more likely to have longer-lasting acute effects on circulating glucose and to elicit greater chronic adaptation which promote better glucose homeostasis (e.g. beneficial changes to body composition, musculature, vascular function, insulin sensitivity) than the same stepping volume accumulated via sporadic incidental lower intensity activity. A stable activity profile may also reflect accumulation of occupational PA which may not confer the same health benefits as leisure time PA. Occupational PA may be consistent across each working day but is typically of a lower intensity, repetitive and often without sufficient recovery time and may also be linked with environmental exposures and occupational stress [28, 43]. Increases in stepping variability per se should likely not be a specific target for behaviour change. However, these examples illustrate that a given stepping volume might be accumulated through different behaviours, with different implications for health, and which are reflected differently in indices of between and within-day stepping variability.

### Strengths and limitations

Strengths of this work include novel focus on stepping and the independent consideration of stepping volume and pattern. Stepping is a fundamental movement behaviour which can be described using simple interpretable metrics reflecting volume and pattern, which lend themselves to self-monitoring within the population and healthcare, and have the potential as the basis of future interventions and public health guidance. Crucially, adjustment for stepping volume revealed that stepping pattern itself may be an important determinant of T2D risk, a finding with important implications.

Nevertheless, this work is not without limitation. To permit analyses of stepping pattern, analyses were limited to participants with ≥ 6 complete days of accelerometer wear which reduced the analytical sample size (Figure S2). Stepping volume was captured in hourly windows. While this is replicable using stepping data from both research and consumer wearables, examination of how steps were accumulated within these windows was not possible which may have masked important differences in how individual stepping events are accumulated [28]. For example previous research suggests that stepping intensity (i.e. cadence) may be important for T2D risk [17]. Advancing analytical processes for accelerometer data will permit future work to determine the independent contributions of frequency, intensity, duration and temporal distribution of stepping events for incident T2D risk. Additionally, while the measure of inter-daily stability captures the uniformity (or not) or daily stepping patterns, we did not additionally examine whether the timing of stepping during the day influenced T2D risk. The activPal3 has excellent analytical validity in a laboratory setting but is more limited in detecting stepping at very low [44, 45] and very high [46] step rates, potentially reducing the range of measured stepping in the present analyses. Like many accelerometer studies, we had limited ability to determine activity type and context, which in future provide important insight into differences in stepping accumulation, and underlying mechanisms for observed differences in health outcomes. The self-report of T2D incidence in The Maastricht study, despite previously showing strong criterion validity, may have introduced some outcome misclassification. Despite the sensitivity of the assessment of T2D [29], it is possible, that reverse causality due to deteriorating health, sub-clinical or occult disease may have affected both T2D incidence and stepping, and have contributed towards the observed associations. Following exclusion of cases within the first 12 months of follow-up, hazard ratios for stepping were strikingly similar, confidence intervals were wider and at times fractionally crossed the threshold to null. While this likely reflects lower statistical power due to the lower number of incident cases we acknowledge that studies of associations of the stepping patterns of T2D risk here with longer follow-up periods and greater diabetes incidence are required. Despite comprehensive sensitivity analyses and adjustment for important confounders, we could not statistically control for all possible influences on T2D risk including key factors such as genetic predisposition and earlier life exposures. As such we acknowledge that associations described may be affected by unmeasured confounding.

### Implications for policy makers and researchers

The proliferation of studies employing wearable movement sensors has permitted insights into dose-response associations between estimates of stepping with incident disease and mortality risk [11, 12]. Learning from the present findings and others, alongside the ubiquity of devices which allow assessment of stepping, may support future integration of stepping metrics within global PA guidance, surveillance and the development and evaluation of interventions [16, 47]. In this study we add important information: that all steps are not equal, and that the pattern in which stepping is accumulated may impact its association with health. These findings bring important considerations for research and may highlight new opportunities for interventions and guidance for improving health through PA. For example, a logical extension of the observation that some stepping patterns may provide more benefit than others is that while increasing daily PA remains an important message, health may be improved by changing stepping patterns without marked changes in stepping volume. This may be particularly important for those for whom increases in stepping volume may be challenging. Specific recommendations regarding how ‘moving differently’ might benefit health may be premature at present. However, continued examination of *how* PA is accumulated, alongside *how much*, should be an important part of further research which underpins development of the next iteration of public health PA guidance, and future behavioural and clinical interventions.

## Conclusions

Our prospective analysis of data from a large cohort study of adults free from T2D suggests that a higher daily stepping volume is associated with substantial reductions in future diabetes risk. Moreover, we have provided novel insights into how the pattern in which a given volume of steps is accumulated within a day and over a week are additionally associated with T2D risk. Accumulating a given volume of steps in a variable pattern which might include larger ‘doses’ of activity may provide the greatest reduction in T2D risk. Further prospective studies are needed to determine how differences in PA accumulation are associated with health outcomes independent of activity volume in order to best inform the next generation of PA guidelines, which are likely to be informed by more device-generated evidence than previous iterations.

## Supplementary Information

Supplementary Material 1: Supplementary Fig. 1 A. Graphical representation of real data from The Maastricht Study illustrating how for a given volume of PA, the way in which PA is accumulated can vary considerably between days of the week and between hours of the day. From Lear et al. 2024 [23]. Dark grey continuous bars represent the average daily step count (all four participants accumulate on average 10 000 steps/day). Lighter grey individual bars represent the absolute daily step count. Black continuous line represents the steps per hour (note the alternate y-axis on right). Participant 1 accumulates approximately 10 000 steps/day on each day with very little variation between different days of the week. Summarizing this participant as achieving on average 10 000 steps/day is therefore fairly accurate. Participant 2 however accumulates the majority of their weekly activity in just 2 days of the week with over 20 000 steps on those days, and much lower levels of activity on the remaining days leading to a higher proportion of activity accumulated in the most active 2 days, and a higher between-day variability. Participant 3 has a fairly continuous pattern of activity accumulation across hours of the day with no large peaks or dips in activity during day time hours, giving a low within- day variability. Participant 4 however has a very fragmented pattern of activity accumulation within each day with some large peaks, followed by dips in activity repeated across hours of the day (shown by the black line) leading to a much higher within-day variability. Supplementary Fig. 1 B. Graphical representation of real data from The Maastricht Study illustrating two participants individual day PA profile (grey lines) superimposed on their average daily PA profile (black line) showing the difference between a high and low inter-daily stability. From Lear et al. 2024 [23]. Participant 5 has a very high inter-daily stability at 0.92 clearly indicated by how well the grey lines reflect the average black line showing that this participant does exactly the same amount of stepping activity at exactly the same times every day of the week. Participant 6 on the other hand has a low inter-daily stability at 0.14 due to obtaining a very different amount of stepping activity at different times on different days of the week; this participant shows little to no routine of activity.

Supplementary Material 2: Supplementary Fig. 2. Participant flow diagram detailing case-wise exclusions from total Maastricht Study cohort through to the final sample for the presented analyses

Supplementary Material 3: Supplementary Fig. 3. Restricted cubic spline for the associations between accelerometer-measured steps/day and incident T2D following exclusion of incident cases recorded during the first 12 months of follow-up. The solid line represents estimated Hazard Ratio for incident T2D and shaded area represents 95% confidence intervals (95% CI). Analyses are based on Cox proportional Hazards Models adjusted for age, sex, educational attainment, smoking status, diet body mass index (BMI), CVD and daily sedentary time

Supplementary Material 4

Supplementary Material 5: Supplementary table S1. Pearsons correlation coefficients for stepping volume and variability metrics. Data are Pearsons correlation coefficients (r). %Active-2days = the proportion of total weekly step count accumulated on the two most active days of the week. BDV = between day step count variability. WDV = within day step count variability. IS = Intra-daily step count stability

## Data Availability

The data that support findings of this study are available from The Maastricht Study, but restrictions apply to the availability of these data, which were used under license for the current study. Data are, however, available from the authors upon reasonable request and with permission of The Maastricht Study management team.

## Abbreviations

PA: Physical activity
MVPA: Moderate to Vigorous Physical Activity
%Active-2days: The proportion of steps accumulated on the two most active days
BDV: Between day variability
IS: Inter-daily stability
WDV: Within Day Variability
SD: Standard deviation
