# Supplementary figures and images for: Not all steps are equal: independent prospective associations of stepping volume and patterns with incident type 2 diabetes mellitus in the Maastricht study
Source: Int J Behav Nutr Phys Act. 2025 Nov 19;22:145. doi: 10.1186/s12966-025-01839-z (PMC12628916; doi:10.1186/s12966-025-01839-z)

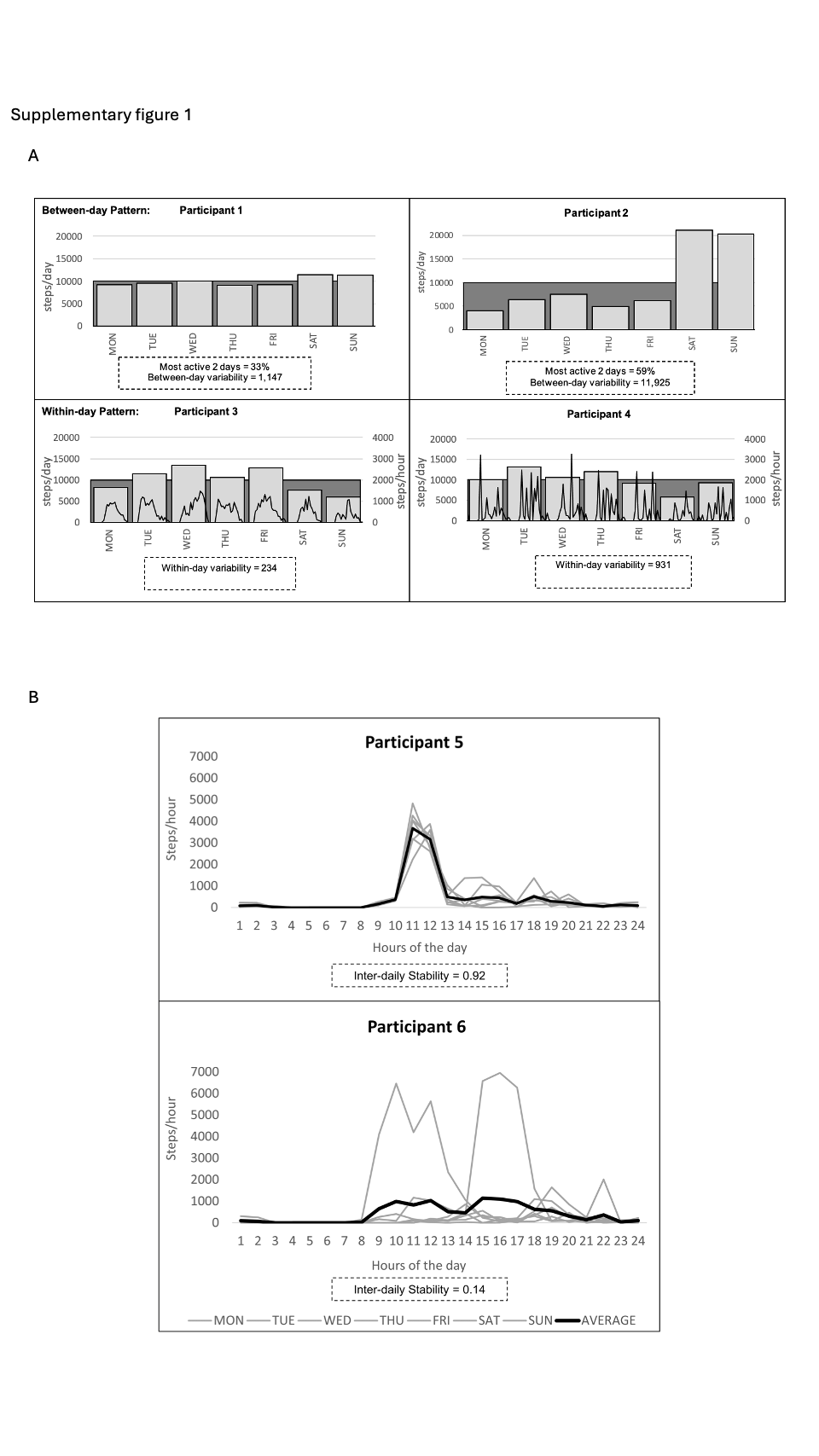

Supplement: Supplementary file 1 — Supplementary Material 1: Supplementary Fig. 1 A. Graphical representation of real data from The Maastricht Study illustrating how for a given volume of PA, the way in which PA is accumulated can vary considerably between days of the week and between hours of the day. From Lear et al. 2024 [23]. Dark grey continuous bars represent the average daily step count (all four participants accumulate on average 10 000 steps/day). Lighter grey individual bars represent the absolute daily step count. Black continuous line represents the steps per hour (note the alternate y-axis on right). Participant 1 accumulates approximately 10 000 steps/day on each day with very little variation between different days of the week. Summarizing this participant as achieving on average 10 000 steps/day is therefore fairly accurate. Participant 2 however accumulates the majority of their weekly activity in just 2 days of the week with over 20 000 steps on those days, and much lower levels of activity on the remaining days leading to a higher proportion of activity accumulated in the most active 2 days, and a higher between-day variability. Participant 3 has a fairly continuous pattern of activity accumulation across hours of the day with no large peaks or dips in activity during day time hours, giving a low within- day variability. Participant 4 however has a very fragmented pattern of activity accumulation within each day with some large peaks, followed by dips in activity repeated across hours of the day (shown by the black line) leading to a much higher within-day variability. Supplementary Fig. 1 B. Graphical representation of real data from The Maastricht Study illustrating two participants individual day PA profile (grey lines) superimposed on their average daily PA profile (black line) showing the difference between a high and low inter-daily stability. From Lear et al. 2024 [23]. Participant 5 has a very high inter-daily stability at 0.92 clearly indicated by how well the grey lines r [file 12966_2025_1839_MOESM1_ESM.tiff]

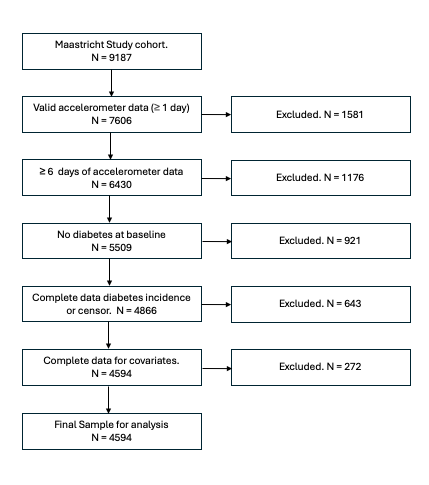

Supplement: Supplementary file 2 — Supplementary Material 2: Supplementary Fig. 2. Participant flow diagram detailing case-wise exclusions from total Maastricht Study cohort through to the final sample for the presented analyses [file 12966_2025_1839_MOESM2_ESM.tiff]

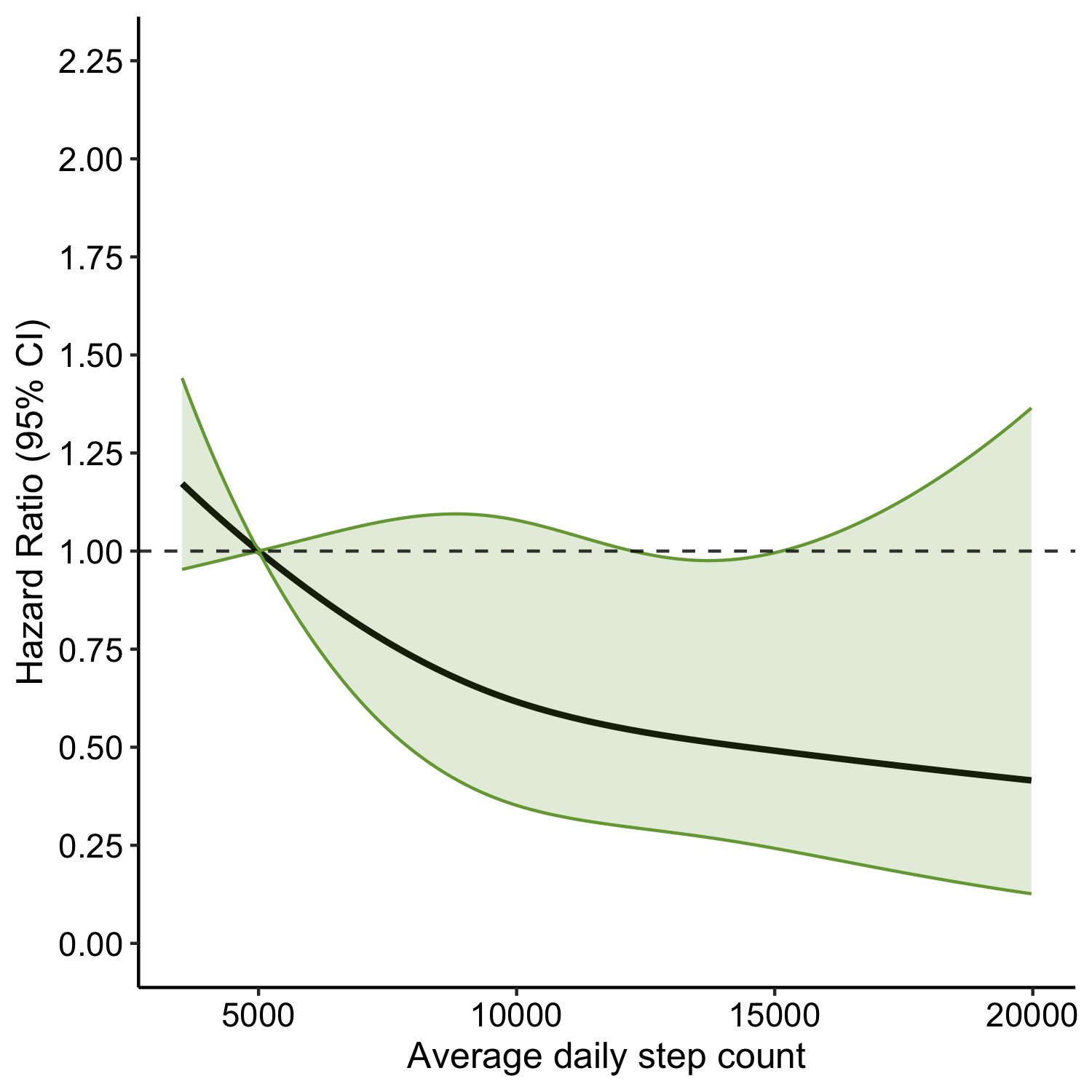

Supplement: Supplementary file 3 — Supplementary Material 3: Supplementary Fig. 3. Restricted cubic spline for the associations between accelerometer-measured steps/day and incident T2D following exclusion of incident cases recorded during the first 12 months of follow-up. The solid line represents estimated Hazard Ratio for incident T2D and shaded area represents 95% confidence intervals (95% CI). Analyses are based on Cox proportional Hazards Models adjusted for age, sex, educational attainment, smoking status, diet body mass index (BMI), CVD and daily sedentary time [file 12966_2025_1839_MOESM3_ESM.tiff]
