# Supplementary material for: Not all steps are equal: independent prospective associations of stepping volume and patterns with incident type 2 diabetes mellitus in the Maastricht study
Source: Int J Behav Nutr Phys Act. 2025 Nov 19;22:145. doi: 10.1186/s12966-025-01839-z (PMC12628916; doi:10.1186/s12966-025-01839-z)
Supplement: Supplementary file 5 — Supplementary Material 5: Supplementary table S1. Pearsons correlation coefficients for stepping volume and variability metrics. Data are Pearsons correlation coefficients (r). %Active-2days = the proportion of total weekly step count accumulated on the two most active days of the week. BDV = between day step count variability. WDV = within day step count variability. IS = Intra-daily step count stability [file 12966_2025_1839_MOESM5_ESM.docx]

**Supplementary Table S1**

|  | **Ave daily steps** | **% 2 most active** | **BDV** | **WDV** | **IS** |
| --- | --- | --- | --- | --- | --- |
| Ave daily steps | 1.0 |  |  |  |  |
| %Active -2days | -0.1 | 1.0 |  |  |  |
| BDV | 0.4 | 0.6 | 1.0 |  |  |
| WDV | 0.7 | 0.7 | 0.4 | 1.0 |  |
| IS | 0.1 | 0.5 | -0.3 | 0.1 | 1.0 |
